# The Role of Metabolic Dysfunction in T-Cell Exhaustion During Chronic Viral Infection

**DOI:** 10.3389/fimmu.2022.843242

**Published:** 2022-03-31

**Authors:** Kehong Zheng, Xiaojun Zheng, Wei Yang

**Affiliations:** ^1^ Department of General Surgery, Zhujiang Hospital, Southern Medical University, Guangzhou, China; ^2^ Research Department of Medical Sciences, Guangdong Provincial People’s Hospital, Guangdong Academy of Medical Sciences, Guangzhou, China; ^3^ Department of Pathology, School of Basic Medical Sciences, Southern Medical University, Guangzhou, China

**Keywords:** chronic viral infection, T-cell exhaustion, metabolism, PD-1, glycolysis

## Abstract

T cells are important components of adaptive immunity that protect the host against invading pathogens during infection. Upon recognizing the activation signals, naïve and/or memory T cells will initiate clonal expansion, trigger differentiation into effector populations and traffic to the inflamed sites to eliminate pathogens. However, in chronic viral infections, such as those caused by human immunodeficiency virus (HIV), hepatitis B and C (HBV and HCV), T cells exhibit impaired function and become difficult to clear pathogens in a state known as T-cell exhaustion. The activation and function persistence of T cells demand for dynamic changes in cellular metabolism to meet their bioenergetic and biosynthetic demands, especially the augmentation of aerobic glycolysis, which not only provide efficient energy generation, but also fuel multiple biochemical intermediates that are essential for nucleotide, amino acid, fatty acid synthesis and mitochondria function. Changes in cellular metabolism also affect the function of effectors T cells through modifying epigenetic signatures. It is widely accepted that the dysfunction of T cell metabolism contributes greatly to T-cell exhaustion. Here, we reviewed recent findings on T cells metabolism under chronic viral infection, seeking to reveal the role of metabolic dysfunction played in T-cell exhaustion.

## Introduction

T cells are crucial components in the pathogen-specific adaptive immune responses. Naïve and/or memory T cells can recognize the specific pathogen *via* T-cell receptors (TCRs) during infection, leading to their robust proliferation and differentiation into effector T cells, which can directly clear pathogens and modulating the local immune response by secreting cytokines (1–5). Following the activation of T cells, dynamic changes were initiated in cellular metabolism, which is known as metabolic reprogramming, to meet their increased energy and anabolic demands. The alteration of metabolism can directly influence the epigenetic status by regulating the activity of histone- and DNA- modifying enzymes, and the production of essential substrates for epigenetic modification (6–8). In addition, metabolic reprogramming can promote the generation of biochemical intermediates (9–13), as well as the biogenesis and oxidative phosphorylation (OXPHOS) of mitochondrial (14–16), which are essential for the proper function of effector T cells. Thus, metabolic reprogramming is fundamental to the proliferation, differentiation and function performance of T cells.

In the context of chronic viral infection, the continuous stimulation of specific pathogens makes effector T cells into a dysfunctional state, which is known as T-cell exhaustion (3, 17–19). The state of exhaustion is characterized by stepwise and progressive loss of T cell function, which is distinct from T cell anergy and senescence (17, 20–23). Although more and more researches start revealing the features of CD4^+^ T cell exhaustion in chronic viral infection (24, 25), in-depth and comprehensive understanding of exhaustion in CD4^+^ T cells are still demanded. In contrast to CD4^+^ T cells, the features of CD8^+^ T cell exhaustion have been much better characterized in chronic viral infection (24, 25). T-cell exhaustion is mainly due to the continuous overstimulation of pathogen, which results in increasing expression of immunosuppressive cytokines and receptor, as well as decreasing the number of help T cells (26). During the state of exhaustion, dynamic changes of metabolism were detected in effector T cell, such as the disturbance of glycolytic metabolism and the reverse of its dependence on fatty acid oxidation (FAO) and OXPHOS for energy generation (26, 27). It has been widely accepted that metabolic dysfunction is tightly associated with the development and progression of T-cell exhaustion (3, 24, 26, 28). In this review, we will focus on the metabolic dysfunction in T-cell exhaustion during chronic viral infection and seeking potential strategies to reverse T-cell exhaustion.

## The Role of Metabolic Reprogramming in T Cell Activation and Function

**Figure 1:**
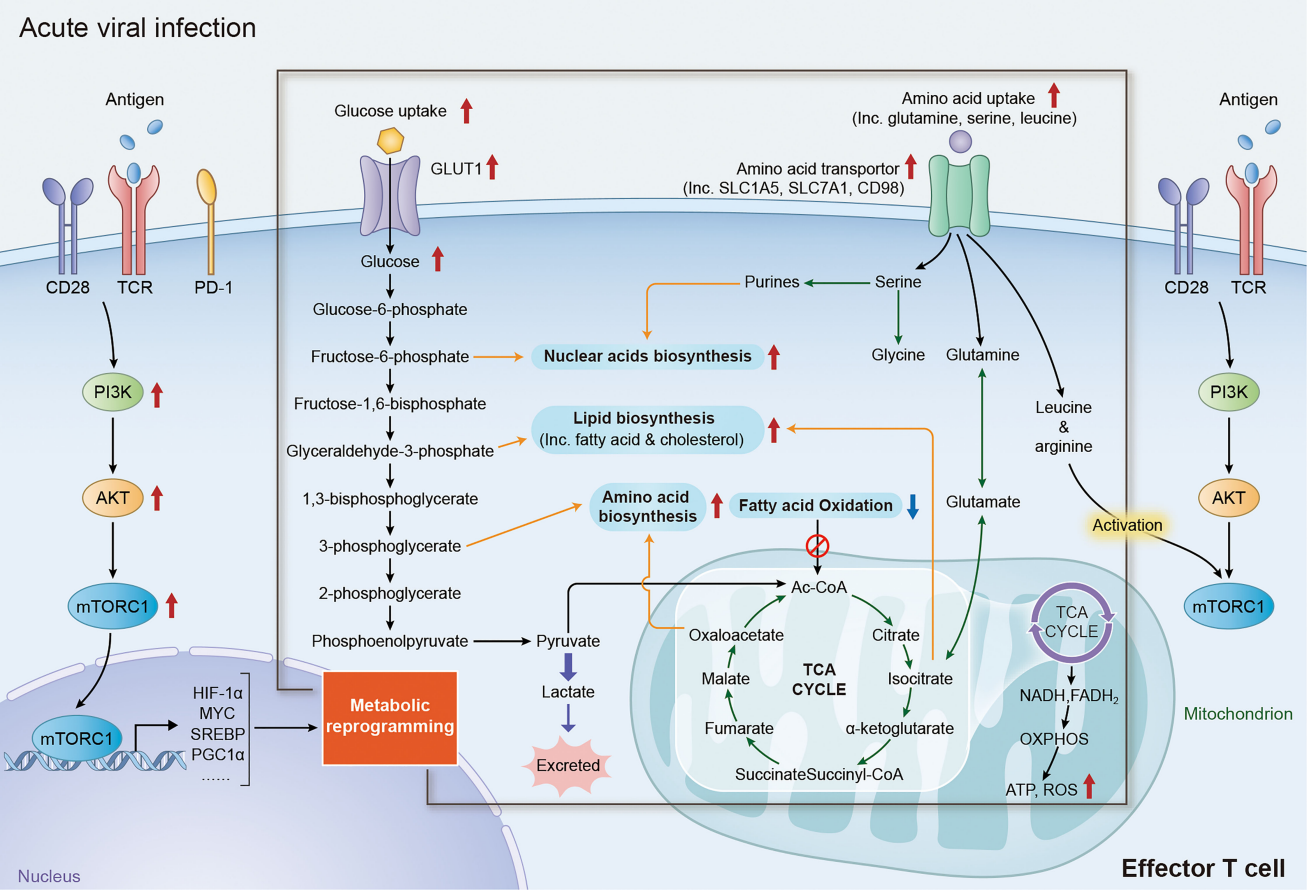
Metabolic reprogramming of effector T cells in acute viral affection. Upon recognition of specific antigen *via* TCR/CD28 receptor, T cells rapidly initiate the signaling of PI3K/AKT/mTORC1 axis and upregulate the expression of its downstream factors including MYC, HIF1α, SREBPs and PGC1α, reprogramming the expression of metabolic transporters and enzymes. Increase expression of glucose transporter family members and glycolytic enzymes dramatically promote the activity of aerobic glycolysis in effector T cells, which not only provide efficient energy generation, but also fueling multi biochemical intermediates that are necessary for nucleotide, amino acid, fatty acid synthesis and mitochondrial function. The reprogramming of amino acid metabolism, especially the changes of glutamine, serine, arginine and leucine metabolism, plays an important role in the activation and functional maintenance of T cells. Augmentation of glutamine metabolism can promote the acetylation reactions or lipid synthesis by acting as an anaplerosis source in the TCA cycle. Increasing level of serine promotes the activity of nuclear acid biosynthesis in effector T cells by fueling the precursor for one-carbon metabolism. In addition, high level of leucine and arginine is required for the full activation of mTORC1 activity in both CD4^+^ and CD8^+^ T cells. TCR, T-cell receptor; PI3K, phosphoinositide 3-kinase; AKT, protein kinase B; mTORC1, mechanistic target of rapamycin complex 1; HIF1α, hypoxia-inducible factor 1-alpha; SREBPs, sterol regulatory element binding proteins; PGC1α, peroxisome proliferator-activated receptor gamma coactivator 1-alpha; TCA, tricarboxylic acid.

Naïve and memory T cells rely primarily on OXPHOS and FAO for energy supply to maintain survival and housekeeping functions (1, 28). Once recognizing of a specific antigen by T cell receptor (TCR), immature T cells will rapidly initiate the activation of phosphoinositide 3-kinase (PI3K)/protein kinase B (AKT)/mechanistic target of rapamycin complex 1(mTORC1) signaling pathway (29), which can subsequently promote the activity of various transcriptional factor including MYC (30, 31), hypoxia-inducible factor 1-alpha (HIF1α) (32, 33), sterol regulatory element binding proteins (SREBPs) (13, 34) and interferon regulatory factor 4 (IRF4) (35, 36) to trigger the reprogramming of metabolism. Engagement of co-stimulatory receptors, such as CD28, is also crucial for the TCR-mediated activation of PI3K/AKT/mTORC1 axis by enhancing the recruitment of PI3K catalytic subunit p110δ (37, 38). It has been well acknowledged that metabolic reprogramming of T cells not only generates efficient energy, but also fuels multiple biochemical intermediates for nucleotide, amino acid, fatty acid synthesis and mitochondrial function (**Figure 1**), which are essential to T cell proliferation, differentiation and proper function performance (6, 8, 39).

The enhancement of aerobic glycolysis (also known as “Warburg Effect”, a distinct form of cellular metabolism, which enable cells getting energies from converting glucose-derived pyruvate to lactate under normoxic conditions, rather than conventionally entering the TCA cycle in mitochondria) is an important events of metabolic reprogramming during CD4^+^ and CD8^+^ T cells activation and functioning (1, 6, 26, 40). TCR/CD28-based activation of PI3K/AKT/mTORC1 signaling can promote the activity of transcriptional factors like MYC and HIF1α, which directly increase the expression of glucose transporter (GLUT) family members and multiple glycolytic enzymes that are vital for facilitating and sustaining the T-cell glycolysis (41–45). Although glycolysis is a relatively inefficient process of ATP production, ATP production by glycolysis is almost 100 times faster than OXPHOS and can sufficiently satisfy the robust energy demand for T cell activation (40, 46). In addition to ATP generation, upregulation of glycolysis also significantly enhances the production of various intermediates including glucose-6-phosphate, glyceraldehyde-3-phosphate and 3-phosphoglcerate, which are crucial precursors for biomolecules synthesis including nucleic acids, lipids and amino acids (1, 6). Moreover, increased pyruvate production could prevent CD4^+^ T cells from apoptosis through maintaining the mitochondrial membrane potential and reactive oxygen species (ROS) level (14). Despite the priority of converting pyruvate into lactate, abundant pyruvate in the cytoplasm produced by the augmentation of aerobic glycolysis can also enter mitochondria for precursor biosynthesis and ATP production, which would also promotes the activation and functions of T cells (1, 6, 42). Beyond the conversion in glycolytic pathways, the alteration of certain glycolytic enzymes during metabolic reprogramming can also regulate the functions of effector T cells through its non-glycolytic functions (16, 47). For example, both CD4^+^ and CD8^+^ T cells significantly reduce the expression and secretion of interferon‐γ (IFN‐γ) in the absence of glucose, which attributes to the reduced binding of glyceraldehyde‐3‐phosphate dehydrogenase (GAPDH) to IFN-γ mRNA (16, 48). The recruitment of enolase-1, a glycolytic enzyme, to the promoter of FoxP3 is essential for the suppressive function of regulatory T (Treg) cells by regulating the transcription level of FoxP3 (47).

The metabolic reprogramming of amino acids, especially changes in glutamine, serine, arginine and leucine metabolism, is important for the activation and function of T cells (49–51). Glutamine is the most abundant amino acid in human body and is an essential nutrient for proliferation (31). Once the pathogen is recognized by TCR in T cells, glutamine uptake is increased by a CD28-dependent upregulation of glutamine transporters. Then, glutamine is further deaminated into glutamate by glutamate synthase (GS) in a MYC-dependent pathway (31, 49). Glutamate can be converted to α-ketoglutarate, which enters the tricarboxylic acid (TCA) cycle as an anaplerosis source to meet the biosynthetic and energy demands for T cells activation, especially under a glucose-restricted condition (52). Glutamate can also promote T cell activation by influencing the metabolism of other biomolecules, including nucleotide, fatty acids and amino acids (53). Beyond the fueling of glutamine for serine biosynthesis, serine uptake in both CD4^+^ and CD8+ T cells are increased during activation. Serine is an important precursor for one-carbon metabolism and plays a crucial role in the proliferation of effector T cells (50). Activation of T cells can also increase the uptake of various neutral amino acids, especially leucine and arginine. Studies have demonstrated that glutamine, leucine and arginine are crucial for the full activation of mTORC1 activity in both CD4^+^ and CD8^+^ T cells (6, 11, 12, 54).

>Fatty acid oxidation (FAO) is a major energy acquisition approach for naïve and/or memory T cells. However, lipid metabolism shifts from catabolism to anabolism in activated T cells to fulfill the boost biosynthetic demands for clonal expansion and differentiation, as well as different T-cell functions (1, 6). TCR-mediated activation of AKT/PI3K/mTORC1 signaling significantly promotes cholesterol and fatty acid synthesis by upregulating the transcription of many lipogenic enzymes in a sterol regulatory element‐binding protein (SREBP)-dependent manner. Deletion of SREBP significantly reduces the clonal expansion and cytokines production of effector T cells during viral infection (13, 55). Studies have demonstrated that the augmentation of cholesterol generation is a hallmark of lipid metabolism during T cell activation and functions, by supporting the synthesis of cellular and organelle membrane (13, 55). Retinoic acid receptor-related orphan receptors (RORs)-induced downregulation of cholesterol-metabolizing enzymes dramatically inhibits the proliferation and survival of CD8^+^ T cells (56). In addition, inhibition of acetyl‐CoA acetyltransferase (Acat) 1 and 2, enzymes involving in cholesterol esterification and storage, can further promote the proliferation and functions of CD8+ T cells by increasing cholesterol levels (57). The enhanced glycolysis and glutamine metabolism in activated T cells also promote fatty acid synthesis by fueling glyceraldehyde-3-phosphate and acetyl-CoA production respectively (1, 53). Notably, metabolic reprogramming of fatty acid synthesis is distinct in the subsets of CD4+ T cells, and tightly linked to their different cell fates (58, 59). Effector CD4^+^ T cells appear to rely more on glucose-derived carbons for fatty acid synthesis, while regulatory T (Treg) cells call for exogenous fatty acid uptake. Moreover, high expression of acetyl‐CoA carboxylase 1, a key enzyme in fatty acid synthesis, can drive the differentiation of CD4^+^ T cells toward Treg cells, other than the Th17 phenotype both *in vitro* and *in vivo* (58, 60).

## Chronic Viral Infection and T-Cell Exhaustion

Generally, adaptive immune responses can successfully clear the virus in acute infections by effector T cells. However, effector T cells fail to clear the pathogens in the context of particular viral infections, including those caused by lymphocytic choriomeningitis virus (LCMV) clone-13, human immunodeficiency virus (HIV), hepatitis B virus (HBV) and hepatitis C virus (HCV), and lead to chronic infection. Persistent infection progressively makes effector T cells progressively into a dysfunctional state that known as T-cell exhaustion, which is characterized by a sequential loss of effector cytokines, upregulation of co-inhibitory receptors, impaired proliferative ability and dysfunctional metabolism (17, 24, 26, 61). Although exhausted CD4^+^ and CD8^+^ T cells share some common features in chronic viral infection, their exhaustion profiles are distinct. Studies in chronic LCMV infection model revealed that CD4^+^ T cells are not merely undergoing exhaustion, but also initiate another pattern of differentiation, which is distinct from exhausted CD8^+^ T cells (62). Since exhausted T cells are heterogeneous, the precise features of exhaustion in CD4^+^ and CD8^+^ T cells are not comprehensively defined, “T-cell exhaustion” is still a broad term for a state which the effector T cells are dysfunction caused by chronic viral infection (63). Following intensive studies will give a better understanding of T-cell exhaustion.

The T-cell exhaustion caused by chronic viral infection is mainly driven by continuous overstimulation of TCR signaling and the subsequently increased expression of many inhibitory and co-inhibitory receptors, including programmed death-1 (PD-1), cytotoxic T lymphocyte antigen-4 (CTLA-4), immunoglobulin domain and mucin domain-containing protein 3 (TIM-3), 2B4 (CD244), lymphocyte activation gene 3 protein (LAG-3) and CD160 (3, 26, 61). The augmentation of PD-1/PD-L1 pathway can block the TCR/CD28 mediated PI3K signaling, dramatically inhibit glycolysis and glutamine utilization in effector T cells and revert its dependent on FAO and OXPHOS for energy generation, to trigger the T-cell exhaustion. Notably, the co-expression of multiple co-inhibitory receptors is essential for PD-1-induced T-cell exhaustion (3). Chronic viral infection can also indirectly induce T-cell exhaustion by altering the function of helper cells like CD4^+^ T cells and dendritic cells (DCs) by secreting cytokines like interleukin (IL)-2 (64). Moreover, some chronic infections such as HBV may alter the metabolism of infected host cells, as well as uninfected adjacent cells. The metabolic alteration of the environment could also lead to the exhaustion of T cells by altering the available nutrients and certain microRNA (miRNAs) levels (26, 65, 66).

## Metabolic Dysfunction in T-Cell Exhaustion

**Figure 2:**
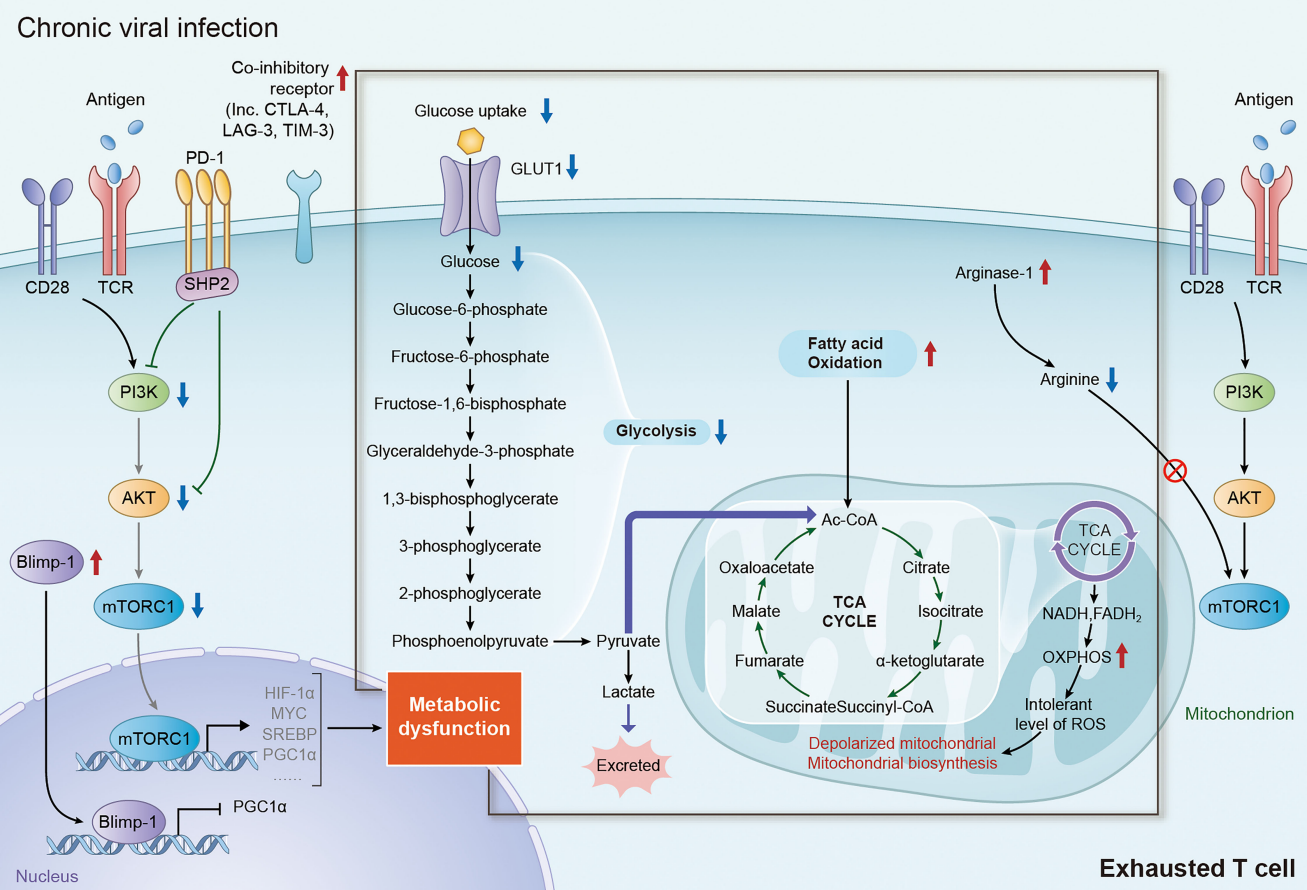
Metabolic alteration of exhausted T cells in chronic viral infection. During chronic viral infection, continuous antigen stimulation *via* TCR signaling significantly increase the expression of multiple inhibitory and co-inhibitory receptors including PD-1, CTLA-4, TIM-3 and LAG-3, which subsequently block the signaling of PI3K/AKT/mTORC1 axis and decrease the expression of its downstream factor, inducing metabolic dysfunction in exhausted T cells. Elevated expression of Blimp-1 in exhausted T cells also induce metabolic dysfunction in exhausted T cells by repressing the expression of PGC1α. It is well accepted that the shifting from glycolysis to FAO for energy generation is an important trigger of T-cell exhaustion. In addition, augmentation of FAO in mitochondria can lead to mitochondrial depolarization, impairment of mitochondrial biosynthesis and production of intolerable level of ROS, which are tightly associated with the dysfunction in exhausted T cells. TCR, T-cell receptor; PI3K, phosphoinositide 3-kinase; AKT, protein kinase B; mTORC1, mechanistic target of rapamycin complex 1; PD-1, programmed death-1; CTLA-4, cytotoxic T lymphocyte antigen-4; TIM-3, immunoglobulin domain and mucin domain-containing protein 3; LAG-3, lymphocyte activation gene 3 protein; PGC1α, peroxisome proliferator-activated receptor gamma coactivator 1-alpha; FAO, fatty acid oxidation; ROS, reactive oxygen species.

It has been well characterized previously that metabolic reprogramming is essential for the activation and function of effector T cells (26, 30, 31, 46). Under chronic viral infection, persistent stimulation of antigen leads to up-regulation of PD-1 and other co-inhibitory receptors in exhausted T cells. Recent studies have highlighted the crucial role of co-inhibitory receptors, especially PD-1, in T cell exhaustion by regulating the transcription of multiple metabolism associated transporters and enzymes, resulting in metabolic alteration of glucose, amino acids and fatty acids (**Figure 2**) (3, 26, 61). Of note, the decrease of glycolysis and mitochondrial respiration in T cells occurs at the early stage in chronic infection, even before T cell dysfunction occurs, suggesting that metabolic dysfunction is an early driver, rather than a consequence, of T cell exhaustion (27, 67, 68). Therefore, targeting metabolism might be an efficient strategy for revising T cell exhaustion in chronic viral infection.

Down-regulation of glycolysis is a key feature of metabolic alteration in exhausted T cells, which occurs at the early stage of T cell exhaustion. Studies with LCMV clone-13 infection model demonstrated that, upon the chronic stimulation of TCR signaling, effector T cells would increase the expression of multiple co-inhibitory receptors, including PD-1 and CTLA-4 (69). The cytoplasmic tail of PD-1 contains an immunotyrosine inhibitory motif (ITIM) and an immunotyrosine switch motif (ITSM), which can attenuate the downstream signal transduction of TCR by recruiting phosphatases, particularly SHP-2 (70, 71). PD-1 ligation would attenuate PI3K/AKT signaling and the activity of mTORC1, block glucose uptake through downregulating Glut1 expression, and inhibit glycolysis by suppressing the expression of hexokinase 2 (HK2), a key enzyme of glycolysis catalyzing the conversion of glucose to glucose-6-phosphate (27, 68, 72). Moreover, CTLA-4, which is also upregulated in exhausted T cells with chronic infection, inhibits glycolysis by a similar mechanism as PD-1 (27, 68).

Augmentation of lipolysis and fatty acid oxidation is another key feature of metabolic alteration in exhausted T cells, but mitochondrial function is compromised. PD-1 ligation in T cells could promote the expression of carnitine palmitoyl transferase 1A (CPT1A), a key regulator of lipid metabolism, to promote FAO in mitochondria in the early stage of exhaustion. Inhibition of CPT1A can reduce mitochondrial respiration by about 50% in early exhausted T cells, suggesting early exhausted T cells switch the energy pathway to FAO when glycolysis is restricted (27, 68). In addition, enhanced expression of PD-1 in exhausted T cells can also promote mitochondrial depolarization by reducing the transcriptional expression of peroxisome proliferator-activated receptor gamma coactivator 1-alpha (PGC1α), a key transcriptional regulator involved in the regulation of energy generation and mitochondrial biogenesis genes, and mitochondrial transcription factor A (mtTFA) (68, 73). Overexpression of PGC1α can promote metabolic fitness by enhanced glycolysis and mitochondrial respiration, and reduce depolarized mitochondria the in early exhausted T cells (17, 27, 68, 73). Recent studies also revealed that continuous TCR stimulation under hypoxia promoted expression of Blimp-1, another transcriptional repressor of PGC1α, which induced the exhaustion of CD8^+^ T cells by repressing PGC1α-dependent mitochondrial reprogramming (74, 75). Despite little is known for the role cholesterol metabolism in chronic infection, it’s well accepted that cholesterol metabolism is crucial in T cell function and exhaustion in cancer. Cholesterol accumulation can promote the expression of co-inhibitory receptors, like PD-1, by increasing endoplasmic reticulum (ER) stress, subsequently initiating the exhaustion of CD8^+^ T cell in tumor microenvironment. Inhibiting ER stress sensor XBP1 or reducing cholesterol can effectively restore the function of effector T cells (76, 77). HCV infection induced a genotype-specific hypocholesterolemia through interfering with late cholesterol synthesis (78), suggesting altered cholesterol level of environment in HCV infection. HIV infection induced SREBP activation could induce TFII-I expression of CD4^+^ T cells. Blocking SREBP by Oxysterols suppress HIV replication in CD4^+^ T cells (79, 80). These results implicate that cholesterol metabolism may play a crucial role in T cell exhaustion during chronic viral infection, which deserves further study.

Recent study found that increasing expression of co-inhibitory receptors also altered the amino acid metabolism in effector T cells. Increased expression of both PD-1 and CTLA-4 significantly reduced the transcriptional expression of key glutamine transporters, SNAT1 and SNAT2, which led to the inhibition of glutamine uptake and glutaminolysis in CD4^+^ T cells (27). Moreover, PD-1 can also inhibit the utilization of the branched chain amino acids (BCAAs), including valine, isoleucine and leucine, which are important for the growth and function of effector T cells by being incorporated into proteins and/or metabolized for energy generation (27, 68).

In addition, a gene-expression profiles study found that a number of energy metabolism and citric-acid cycle associated genes were transcriptionally downregulated in exhausted CD8+ T cells, including Acas2l, Sdha, Adcy7, Pdha1, and Acadm, suggesting more metabolic pathway may involve in the energy depression in exhausted T cells during chronic infection (81). However, mechanisms that how these metabolism related genes involved in T cell exhaustion still require further investigation.

## Conclusion

T-cell exhaustion is a broad definition of dysfunctional effector T cells in chronic viral infection, since the features of, as well as the pathways to, T-cell exhaustion are still not fully understood. Over the past decades, studies have demonstrated the importance of metabolic reprogramming in the activation and function of effector T cells. Although the characteristic of metabolic alterations in exhausted T cells is still not comprehensively investigated, recent studies highlighted the crucial roles of co-inhibitory receptors mediated in glycolytic and lipidic metabolic dysfunction during T-cell exhaustion, suggesting co-inhibitory receptor could be a promising target to improve anti-virus function of T cells. In addition to co-inhibitory receptors, whether there are other mechanisms regulating T cell metabolism in chronic infection still deserves more researches. Since metabolic dysfunction in exhausted T cells is complicated and heterogeneous, it requires more studies to clarify the features, as well as the underlying mechanisms, of metabolic dysfunction in exhausted T cells, including the alterations of glucose, lipid and amino acid metabolism, aiming to provide new insights with potential and promising target in anti-virus immunotherapy.

## Author Contributions

All authors listed have made a substantial, direct and intellectual contribution to the work, and approved it for publication.

## Funding

The work has also been funded by National Key R&D Program of China (No. 2018YFA0800404 to WY), National Natural Science Foundation of China (NSFC, No. 81822036 and 31770931 to WY, No. 82103399 to KZ, No. 31800730 to XZ), Guangdong Natural Science Funds for Distinguished Young Scholar (No. 2017A030306030 to WY),Guangdong Basic and Applied Basic Research Foundation (No. 2020A1515010212 to KZ, No. 2020A1515011246 to XZ), Science and Technology Program of Guangzhou (No. 202102020903 to KZ).

## Conflict of Interest

The authors declare that the research was conducted in the absence of any commercial or financial relationships that could be construed as a potential conflict of interest.

## Publisher’s Note

All claims expressed in this article are solely those of the authors and do not necessarily represent those of their affiliated organizations, or those of the publisher, the editors and the reviewers. Any product that may be evaluated in this article, or claim that may be made by its manufacturer, is not guaranteed or endorsed by the publisher.

## Abbreviations

HIV, human immunodeficiency virus; HBV, hepatitis B; HCV, hepatitis C; LCMV, lymphocytic choriomeningitis virus; PD-1, programmed death-1; CTLA-4, cytotoxic T lymphocyte antigen-4; YIM-3, immunoglobulin domain and mucin domain-containing protein 3; LAG-3, lymphocyte activation gene 3 protein; PD-L1, programmed death ligand-1; TCR, T-cell receptor; OXPHOS, oxidative phosphorylation; FAO, fatty acid oxidation; PI3K, phosphoinositide 3-kinase; AKT, protein kinase B; mTORC1, mechanistic target of rapamycin complex 1; TCA, tricarboxylic acid; HIF1α, hypoxia-inducible factor 1-alpha; SREBP, sterol regulatory element binding proteins; IRF4, interferon regulatory factor 4; GLUT, glucose transporter; ROS, reactive oxygen species; IFN‐γ, interferon‐γ; GAPDH, glyceraldehyde‐3‐phosphate dehydrogenase; Treg, regulatory T; GS, glutamate synthase; RORs, Retinoic acid receptor-related orphan receptors; Acat, acetyl‐CoA acetyltransferase; DCs, dendritic cells; IL, interleukin; miRNAs, microRNAs; PGC1α, peroxisome proliferator-activated receptor gamma coactivator 1-alpha; ITIM, immunotyrosine inhibitory motif; ITSM, immunotyrosine switch motif; Blimp-1, PR domain zinc-finger protein 1.
